# A Case Report of Valacyclovir-Associated Neurotoxicity in End-Stage Renal Disease: A Rare but Preventable Side Effect

**DOI:** 10.7759/cureus.45737

**Published:** 2023-09-21

**Authors:** Amber Y Bo, Xin Ran Li, Balpreet Kaur

**Affiliations:** 1 Internal Medicine, Medical College of Wisconsin, Milwaukee, USA

**Keywords:** end-stage renal disease, genital herpes, altered mental status, hsv-2, esrd, van, valacyclovir-associated neurotoxicity

## Abstract

Neurotoxicity can develop as a side effect of valacyclovir in patients with renal disease, especially without a renally adjusted dose. We present a 56-year-old female with end-stage renal disease (ESRD) on hemodialysis (HD) who presented to the emergency room (ER) with agitation and confusion and was found to have valacyclovir-associated neurotoxicity (VAN). Five days prior, she had been prescribed the standard treatment of 500 mg valacyclovir twice daily for three days for herpes simplex virus-1 (HSV-1); however, her creatinine clearance was low enough to require a renally adjusted dose. Her condition was worsened from missing a dialysis session due to acute confusion. She was treated with three days of hemodialysis sessions. Improvement in mentation and agitation was observed after the second day of hemodialysis, and a complete resolution of symptoms and return to cognitive baseline occurred two days later. There are reports of daily hemodialysis shortening the neurotoxicity period and resulting in a faster return to normal mentation. This case is important as the dose of valacyclovir must be adjusted in those with kidney disease.

## Introduction

The prodrug, valacyclovir, is converted in vivo to acyclovir, a nucleoside analog that competitively inhibits viral DNA polymerase. This medication is the first-line treatment of herpes simplex virus-1 (HSV-1), genital herpes (herpes simplex virus-2 (HSV-2)), and varicella zoster virus (VZV). Valacyclovir has 55% greater bioavailability than acyclovir and is preferred due to requiring less frequent doses to achieve the same therapeutic level. The antiviral is renally cleared, and dose adjustment is recommended in patients with decreased creatinine clearance. The most common side effects include headache, nausea, and abdominal pain. While generally well-tolerated, patients with renal impairment are particularly vulnerable to valacyclovir-associated neurotoxicity (VAN), a rare central nervous system (CNS) adverse reaction that manifests as agitation, hallucinations, and confusion [1]. The risk of VAN is heightened when patients with renal impairment do not receive an appropriately reduced dose. We present a case of VAN in a patient with end-stage renal disease (ESRD) treated for recurrent HSV-2.

## Case presentation

A 56-year-old female with a history of ESRD on hemodialysis (HD) presented with acute on subacute altered mental status. The patient was prescribed valacyclovir at the standard dose of 500 mg twice daily for three days for recurrent genital herpes. She presented to the emergency department (ED) after three doses of valacyclovir in 48 hours with a headache, vomiting, abdominal pain, dizziness, tingling in the extremities, hallucinations, and a decreased ability to walk. She had a past medical history of atrial fibrillation on warfarin, type II diabetes mellitus, hypertension, non-ischemic cardiomyopathy, secondary hyperparathyroidism, obstructive sleep apnea, and chronic respiratory failure on oxygen. The patient was not taking any medications concerning for drug-drug interactions with valacyclovir.

**Figure 1:**
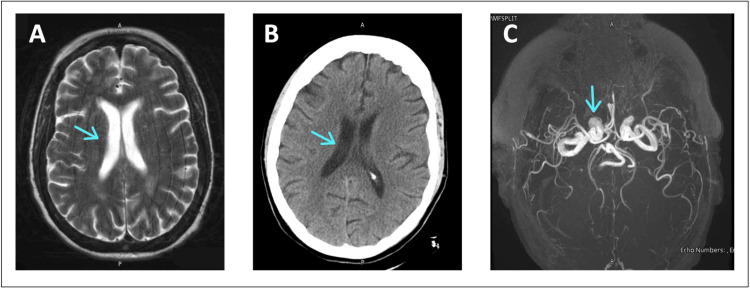
MRI, CT, and MR Angiogram of the Head (A) MRI of the brain without contrast, AX T2 series: mild chronic ischemic changes with no acute intracranial process evident. (B) Axial CT of the head without contrast: no acute intracranial process and no significant interval change. (C) 3D TOF MR angiogram of the circle of Willis without contrast: unremarkable MRA of the circle of Willis. All arrows indicate areas of normal intracranial processes with no acute changes. MRI: magnetic resonance imaging, CT: computed tomography, MRA: magnetic resonance angiography, TOF: time of flight

The patient was afebrile and hemodynamically stable. On physical examination, there were no focal neurological deficits, and radiography of the head (magnetic resonance imaging (MRI), computed tomography (CT), and MR angiogram) was unremarkable (Figure 1). Blood work showed no leukocytosis, and lumbar puncture was not performed due to an absence of nuchal rigidity and low suspicion for meningitis. She had exceeded the recommended ESRD-adjusted maximum dose of valacyclovir (500 mg valacyclovir daily for three days), and her symptoms were suspected to be valacyclovir side effects. The offending drug was stopped, and she was discharged to follow up with her primary care physician the next day.

**Figure 2:**
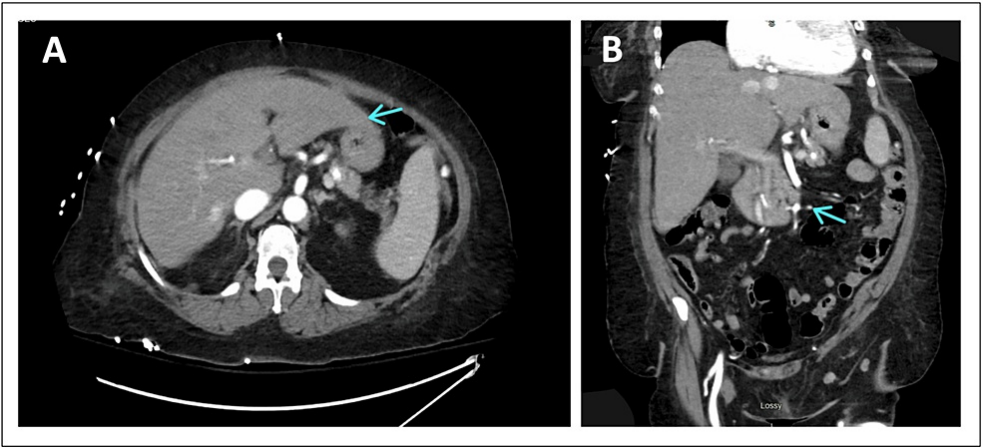
CT of the Abdomen and Pelvis (A) Axial view. (B) Coronal view. No acute findings in the abdomen or pelvis were found. Both arrows indicate areas of normal abdominal findings with no acute changes. CT: computed tomography

Three days later, she presented to the ED again with worsening mental status, and new myoclonus and unintelligible speech. She had missed dialysis the day before due to confusion. Her mother noted that the patient’s memory was fluctuating, and she was refusing to eat and walk and was combative toward family members. She was hypertensive but afebrile, and laboratory findings indicated hyperkalemia (6.4 mEq/L), elevated creatinine (15.21), and anion gap metabolic acidosis. CT of the abdomen and pelvis was unremarkable (Figure 2). There was no nuchal rigidity, and no lumbar puncture was performed due to low suspicion for meningitis.

**Figure 3:**
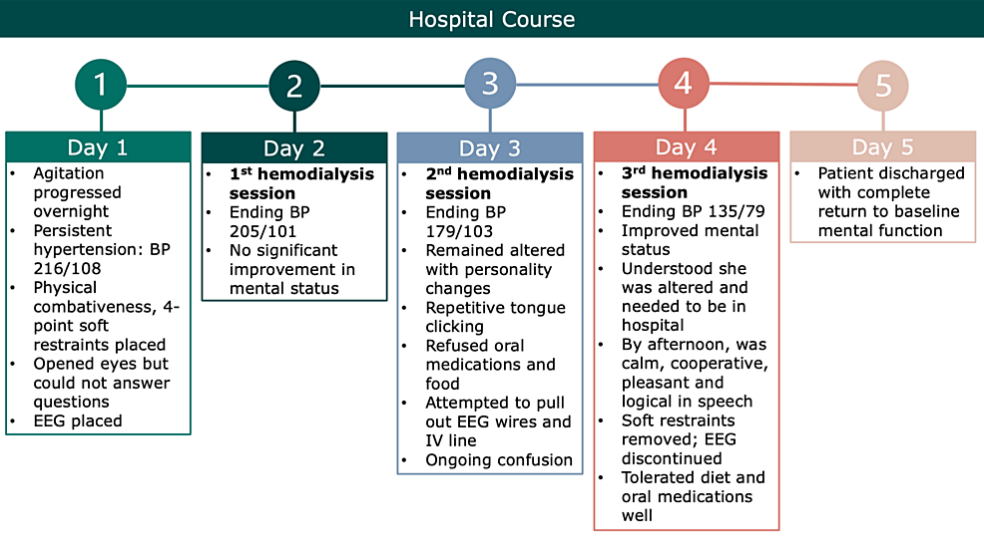
Hospital Course The figure shows the hospital course that includes details of the management of our patient and a timeline of the resolution of her symptoms. BP: blood pressure, EEG: electroencephalogram

The patient was admitted for valacyclovir-induced encephalopathy, and her course was complicated by refusing oral intake and becoming nonverbal with repetitive tongue clicking and combative behaviors. The laboratory finding for acyclovir serum level was not available. Continuous electroencephalogram (EEG) showed diffuse slowing, consistent with encephalopathy or a reversible toxic metabolite state. She received hemodialysis for three consecutive days with significant improvement in mentation after two sessions. On the third morning, she was alert and oriented with pleasant and logical speech. The patient was discharged home after five days of hospitalization with a complete resolution of symptoms (Figure 3).

## Discussion

Valacyclovir is an antiviral prodrug that is converted in the body to its active metabolite, acyclovir. With its increased bioavailability and less frequent dosing, valacyclovir quickly became the preferred treatment of initial and recurrent genital and mucocutaneous herpes simplex infections (HSV-1 and HSV-2) and varicella zoster virus (VZV) after its inception in 1995 [1]. The antiviral is generally well-tolerated with headache, nausea, or abdominal pain as the most common side effects.

**Figure 4:**
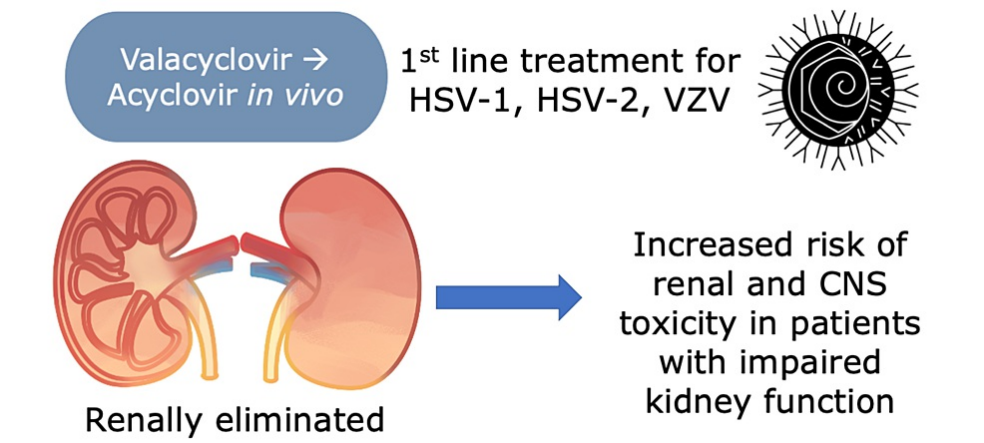
Simple Diagram of Valacyclovir Metabolism and Adverse Events The diagram is the author’s own creation. HSV: herpes simplex virus, VZV: varicella zoster virus, CNS: central nervous system

However, in patients with renal impairment, there is an increased risk of valacyclovir-associated neurotoxicity (VAN), a rare CNS side effect of delirium, confusion, agitation, and encephalopathy [2]. While a small percentage of valacyclovir is eliminated in the feces, the majority (89%) is converted to acyclovir, which is metabolized by the kidneys (Figure 4) [3]. This increases the risk of adverse events in patients with renal impairment as they are unable to clear the metabolized acyclovir effectively. In patients with ESRD, the half-life of the drug increases from three hours to 14 hours [2,3]. It is crucial for patients undergoing hemodialysis to attend their sessions to ensure effective drug clearance.

The standard dose for a recurrent episode of genital herpes is 500 mg twice a day for three days; however, in a patient with chronic kidney disease and a creatinine clearance under 10 mL/minute/1.73 m^2^, the dose is reduced significantly to 500 mg daily [4]. Dose adjustment is necessary for patients with chronic kidney disease, especially those with an estimated creatinine clearance below 30 mL/minute/1.73 m^2^ [4]. Our patient had ESRD and a creatinine clearance of 4.6 mL/minute, which required a reduced dose of 500 mg daily, yet she was prescribed the standard dosing, increasing her susceptibility to neurotoxicity.

A hemodialysis session removes 33% of the valacyclovir, and our patient missed dialysis after the onset of altered mental status, exacerbating the accumulation of valacyclovir in her system. Impairment of consciousness is reported to develop within 24-48 hours after peak serum concentration [4].

Severe neurotoxicity in dialysis patients has been reported at acyclovir doses of 800 mg twice daily, which is approximately 500 mg valacyclovir [5]. It is likely that our patient already had VAN on her first presentation to the ED, while the offending drug was stopped, the severity of her symptoms was unrecognized, and appropriate treatment with hemodialysis was delayed. Neurotoxicity is best managed with hemodialysis, which rapidly shortens the neurotoxicity period, and most patients have a complete resolution of symptoms in 2-7 days [4].

The recognition of VAN has improved in recent years with increased case reports and studies. In one case, a 77-year-old ESRD patient presented with confusion and hallucination after ingesting a high dose of valacyclovir 3 grams per day for shingles [6]. Another case presented an ESRD patient taking a higher dose of 4 grams per day presenting with pseudobulbar affect [7]. In addition to mild to moderate neurotoxicity, valacyclovir also manifests as severe altered mental status and seizures. A 24-year-old ESRD patient presented with tonic-clonic seizure and status epilepticus resistant to lorazepam and phenytoin [3]. A systematic review of 97 clinical cases and case series analyzing a cohort of 119 patients found that while acyclovir and valacyclovir neurotoxicity is still a rare side effect, it most commonly presents in those over 65 years old and in ESRD patients. The time of onset is usually after three days of treatment with valacyclovir, and complete recovery of mentation typically occurs within seven days [8].

With increasing recognition of VAN, one population-based retrospective study examined patients with ESRD on dialysis with individuals with normal renal function. The study found that the incidence of altered mental status of valacyclovir users on hemodialysis (HD) and peritoneal dialysis (PD) was higher than that of non-users [9]. However, ESRD patients on adjusted valacyclovir doses and receiving regular, timely dialysis without missing sessions did not have VAN side effects [9]. Patients on hemodialysis taking a valacyclovir dose of less than 500 mg three times per week and patients on peritoneal dialysis taking a dose of less than 500 mg per day did not have altered mental status [9]. VAN can be preventable if patients with ESRD are on renally adjusted valacyclovir doses and monitored closely while on medications.

Acyclovir has low protein binding and a low volume of distribution, and as such, its elimination may be enhanced with hemodialysis. Prior reports describe the removal of 30%-60% of the drug during four-hour hemodialysis sessions; however, it is rarely indicated as patients often do well with supportive care alone [2,9]. Hemodialysis has been used successfully in patients with severe neurotoxicity from acyclovir and valacyclovir and should be used in these severe cases to enhance the elimination of the offending drug [2,3].

## Conclusions

In summary, we present an important case of how rapidly valacyclovir-associated neurotoxicity (VAN) can develop in a patient with ESRD without a renally adjusted dose. While VAN is considered rare, it has been documented in the literature. Our aim is to enhance its prompt recognition in clinical practice. Additionally, we want to highlight how patients can become vulnerable to missing scheduled dialysis and exacerbating their neurotoxicity if they experience more severe manifestations of altered mental status.

Valacyclovir is a highly effective and relatively well-tolerated treatment for HSV-1, HSV-2, and VZV, but clinicians must have a high degree of suspicion to investigate recent valacyclovir use in patients with kidney impairment who present with new behavioral changes. In particular, patients with ESRD on hemodialysis have a depressed creatinine clearance and are particularly susceptible to valacyclovir-associated neurotoxicity. Adhering to renally adjusted doses is recommended for clinicians to prevent drug accumulation and toxicity. There could be a potential benefit to implementing warnings in the electronic medical record for valacyclovir prescriptions in patients undergoing hemodialysis. It is also imperative to recognize when patients require more immediate treatment, including hospitalization and consecutive hemodialysis. Hemodialysis, as seen in our patient’s hospital course and in the literature, has been shown to be a safe and effective method to resolve the symptoms of VAN. This case highlights the importance of a renally adjusted dose, prompt discontinuation of medication when symptoms occur, and immediate treatment with hemodialysis if neurotoxicity is severe.
